# Identification and Characterization of Nnematicidal Volatile Organic Compounds from Deep-Sea *Virgibacillus dokdonensis* MCCC 1A00493

**DOI:** 10.3390/molecules25030744

**Published:** 2020-02-09

**Authors:** Dian Huang, Chen Yu, Zongze Shao, Minmin Cai, Guangyu Li, Longyu Zheng, Ziniu Yu, Jibin Zhang

**Affiliations:** 1 State Key Laboratory of Agricultural Microbiology, National Engineering Research Center of Microbe Pesticides, College of Life Science and Technology, Huazhong Agricultural University, Wuhan 430070, China; hui8229hd@163.com (D.H.); yuchen921204@163.com (C.Y.); cmm114@mail.hzau.edu.cn (M.C.); ly.zheng@mail.hzau.edu.cn (L.Z.); yz41@mail.hzau.edu.cn (Z.Y.); 2 Key Laboratory of Marine Biogenetic Resources, Third Institute of Oceanography, Ministry of Natural Resources, Xiamen 361005, China; shaozz@163.com (Z.S.); mccc_ligy@163.com (G.L.)

**Keywords:** *virgibacillus dokdonensis*, *Meloidogyne incognita*, volatile organic compound, fumigant, attraction, repellent, integrated strategy

## Abstract

Root-knot nematode diseases cause severe yield and economic losses each year in global agricultural production. *Virgibacillus dokdonensis* MCCC 1A00493, a deep-sea bacterium, shows a significant nematicidal activity against *Meloidogyne incognita* in vitro. However, information about the active substances of *V. dokdonensis* MCCC 1A00493 is limited. In this study, volatile organic compounds (VOCs) from *V. dokdonensis* MCCC 1A00493 were isolated and analyzed through solid-phase microextraction and gas chromatography–mass spectrometry. Four VOCs, namely, acetaldehyde, dimethyl disulfide, ethylbenzene, and 2-butanone, were identified, and their nematicidal activities were evaluated. The four VOCs had a variety of active modes on *M. incognita* juveniles. Acetaldehyde had direct contact killing, fumigation, and attraction activities; dimethyl disulfide had direct contact killing and attraction activities; ethylbenzene had an attraction activity; and 2-butanone had a repellent activity. Only acetaldehyde had a fumigant activity to inhibit egg hatching. Combining this fumigant activity against eggs and juveniles could be an effective strategy to control the different developmental stages of *M. incognita*. The combination of direct contact and attraction activities could also establish trapping and killing strategies against root-knot nematodes. Considering all nematicidal modes or strategies, we could use *V. dokdonensis* MCCC 1A00493 to set up an integrated strategy to control root-knot nematodes.

## 1. Introduction

Plant-parasitic nematodes (PPNs) cause an annual loss of over $150 billion in world crops [1]. Root-knot nematodes (RKNs) are obligate root parasites that infest more than 5000 plant species worldwide. *Meloidogyne incognita* is a RKN and one of the most severe PPNs worldwide [2,3], especially in tropical and subtropical agricultural areas [2,4,5,6]. This pathogen enters roots and establishes a feeding site, resulting in the formation of a large gall in a susceptible host [7]. These large galls impair the ability of plants to uptake water and nutrients, and they can lead to symptoms, such as wilting, stunting, chlorosis, and ultimately yield loss [8]. Infection by *Meloidogyne* spp. may predispose a plant to secondary pathogens [7,9]. Controlling *Meloidogyne* spp. is sometimes difficult because of their extensive host range, short life cycle, high reproductive rate, and endoparasitic nature [10]. *Meloidogyne* spp. are also difficult to control with a single control method [11]. Several management strategies, including crop rotations, developing and planting resistant varieties, use of chemical nematicides, and biological and physical control measures, can be applied to control RKNs [12,13,14]. Among them, chemical nematicides have been generally used because they are efficacious, can be easily applied, and have a rapid onset [15].

Although chemical nematicides are usually more effective than other strategies, they have caused significant environmental problems because of their toxic residues. For example, bromomethane, an effective soil fumigant, is no longer used because of its destructive potential to the stratospheric ozone [16]. Dibromochloropropane, known as an effective organochlorine nematicide, has been banned since 1979 because of its mutagenicity, carcinogenicity, and reproductive effects on humans [17]. Therefore, developing environment-friendly alternatives is urgently needed for PPN control.

PPNs usually exist in soil and are subjected to infection by indigenous bacteria and fungi in soil, thereby providing the possibility of using microorganisms to control PPNs [18]. Many microorganisms and their metabolites have been extensively studied, and they have shown great potential for the biological control of nematodes [19,20,21]. Volatile organic compounds (VOCs) obtained from terrestrial organisms have significant biological activities. Gu et al. evaluated the VOCs produced by 200 isolates of soil bacteria in in vitro experiments. Most soil bacteria show a nematicidal activity against PPNs. The detected VOCs include alcohols, aldehydes, ketones, alkenes, and ethers [22].

VOCs are small volatile compounds that can be fumigated at certain temperature and pressure to exert their insecticidal or bacteriostatic effects [23]. Microorganisms can release volatile substances in soil or other growth matrices, and the fumigation of these substances can be used to prevent and control plant diseases [24]. Cheng [25] et al. found that volatile substances produced by *Paenibacillus polymyxa* can control RKNs in many ways. Zhai [26] et al. detected seven volatile substances in the fermentation broth of *Pseudomonas putida* 1A00316; among them, 2-undecanone has shown a strong nematicidal activity. VOCs have a certain toxic effect on plant pathogens and can attract nematode natural enemies. As activation signal molecules of plant resistance-related genes, VOCs can enhance the resistance of plants to pathogens by activating the plant hormone-dependent signal pathway [27]. VOCs are generally less toxic to humans and livestock; hence, the development and application of online biological control agents have great potential. 

Oceans cover more than 70% of the Earth’s surface, and this proportion represents more than 95% of the biosphere by volume [28]. Deep-sea bacteria are abundant microbial resources found in these water bodies. Marine microorganisms have special living conditions, so they can often produce various active compounds with functions and structures that may be different from those found in terrestrial organisms. We previously reported the special antibacterial activity of *Virgibacillus dokdonensis* against *Xanthomonas oryzae* pv. *oryzae* [29]. The active compound 1-deoxy-*N*-acetylglucosamine was also extracted and identified. To our knowledge, few studies have described the nematicidal activity of volatiles from marine microorganisms and their potential use as substitutes for highly toxic chemical nematicides. *Virgibacillus dokdonensis* MCCC 1A00493 is originally isolated from deep-sea polymetallic nodules in the Eastern Pacific Ocean and exhibits a strong nematicidal activity against RKNs. In this study, the VOCs produced by MCCC 1A00493 and their multiple active mechanisms on *Meloidogyne incognita* were investigated. 

## 2. Results

### 2.1. Contact and Fumigant Nematicidal Activities of V. dokdonensis’s Fermentation Supernatant against M. incognita J2s

**Figure 1:**
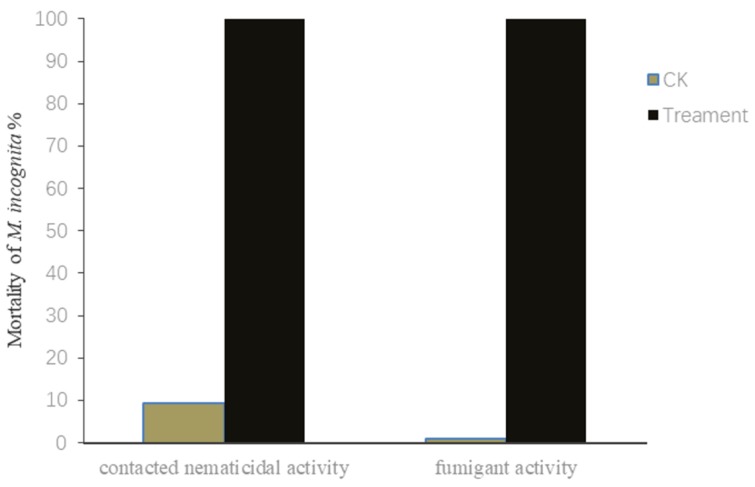
Nematicidal activity of *V. dokdonensis* (Treatment) and control group (CK) against *M. incognita*.

The fermentation supernatant of *V. dokdonensis* MCCC 1A00493 (OD_600_ = 1.7) showed a high activity against *M. incognita* in vitro. The mortality rate was 100% (24 h) when the nematode juveniles were exposed to the supernatant of MCCC 1A00493 at five times of dilution (Figure 1). The fumigant activity test also revealed that the6 MCCC 1A00493 culture could kill nematodes by producing volatiles. The nematicidal activity of this strain was 100% at 24 h (Figure 1).

### 2.2. Identification of the VOCs of V. dokdonensis

**Figure 2:**
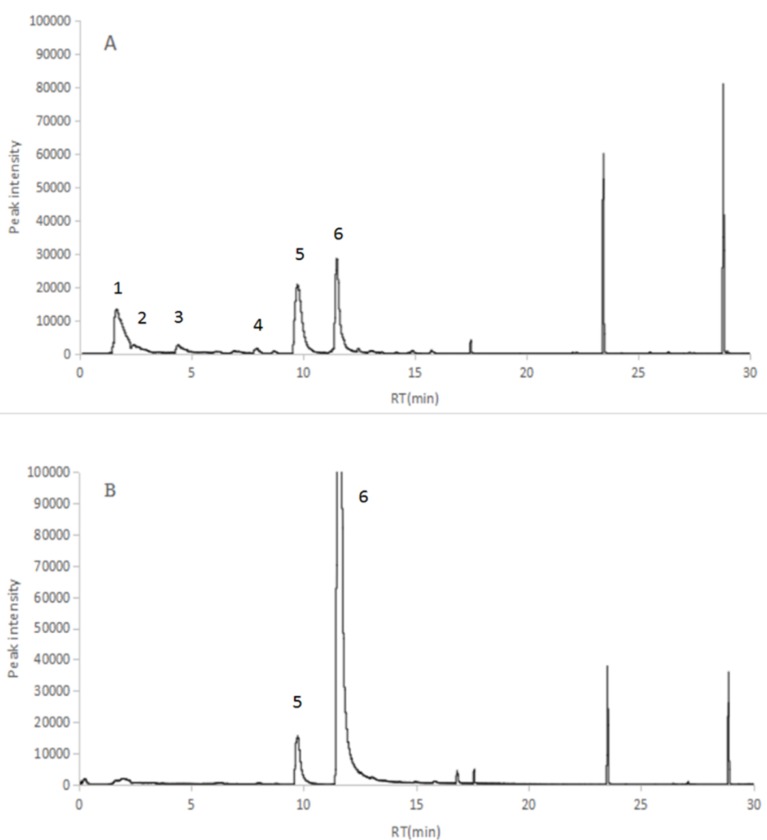
Chromatograms of VOCs in the TIC mode via CAR/DVB extraction. (**A**) Culture of *V. dokdonensis* 1A00493; (**B**) 2216E medium.

Six peaks from the bacterial culture were observed in the total ion current chromatograms, and two peaks were detected in the 2216E medium through GC-MS analysis (Figure 2). After these peaks were compared with the mass spectrum of the substance from the GC-MS system data bank (NIST 08 library), four kinds of volatiles (acetaldehyde, 2-butanone, dimethyl disulfide, and ethylbenzene) produced by the bacterium and two kinds of volatiles (2,5-dimethyl pyrazine and benzaldehyde) in the 2216E medium were identified (Table 1). The MCCC 1A00493 fermentation broth produced mixtures of VOCs and killed nematode juveniles. Four compounds, namely, acetaldehyde, 2-butanone, dimethyl disulfide, and ethylbenzene, were purchased for further experiments.

### 2.3. Nematicidal Activity of VOCs against M. incognita J2s

**Figure 3:**
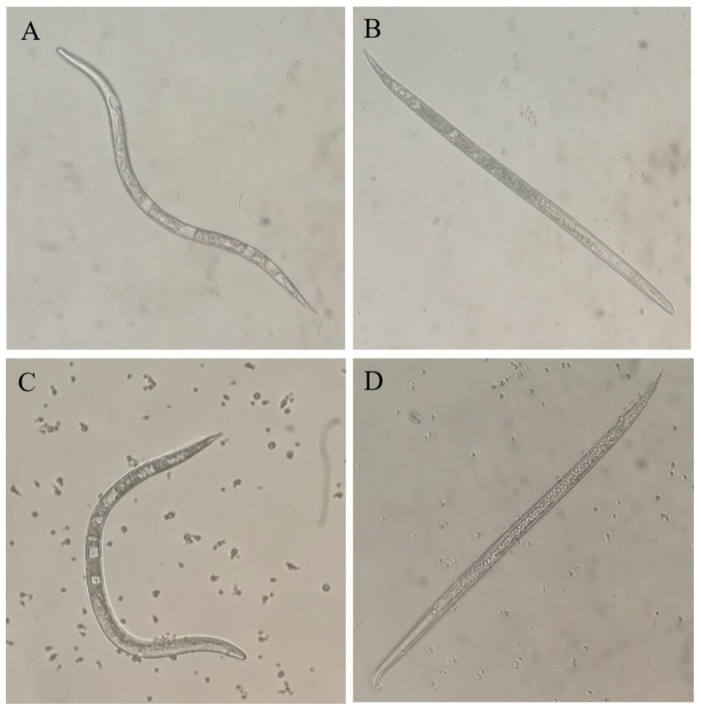
Morphological variations in *M. incognita* J2s after acetaldehyde and dimethyl disulfide treatments. (**A**) Treated with H_2_O for 24 h; (**B**) treated with 10 mg/mL acetaldehyde for 24 h; (**C**) treated with 0.3% Tween 20 for 24 h; (**D**) treated with 10 mg/mL dimethyl disulfide for 24 h.

The four VOCs produced by the bacterium were taken as the nematicidal candidates, and their nematicidal efficacy was measured in vitro by using commercial compounds as described before (Table 1). Two VOCs (acetaldehyde and dimethyl disulfide) from the four tested candidates exhibited strong nematicidal activities against juveniles at a concentration of 1 mg/mL. After being exposed to acetaldehyde and dimethyl disulfide for 24 h, the nematodes died, their body shape straightened, and the inner tissues were destroyed entirely (Figure 3). After acetaldehyde and dimethyl disulfide treatments were administered, the RKNs presented a stiff and dead state, and the intestinal tissue in the body cavity was not clear and was suspected to be damaged. The nematicidal activity of acetaldehyde had EC_50_ of 141.4 µg/mL at 6 h and below 10 µg/mL at 24 h. The nematicidal activity of dimethyl disulfide had EC_50_ of 139.1 µg/mL at 24 h. The EC_50_ values of 2-butanone and ethylbenzene were not evaluated because of their low nematicidal activity at 1 mg/mL (Table 2).

### 2.4. Fumigant Activity of VOCs against Juveniles and Eggs

The fumigant activity of volatiles against juveniles was tested in 96-well plates. At 10 mg/mL, only acetaldehyde had a fumigant activity against J2 (Figure 3), whereas the mortality of the three other volatiles was less than 10%. Even when the concentration of acetaldehyde was reduced to 1 mg/mL, its fumigation effect was still obvious, and the mortalities of the nematodes were 70.1% at 6 h and 98.0% at 24 h (Table 3).

We also tested acetaldehyde’s fumigant activity to inhibit egg hatching. The three other compounds were not further tested because only acetaldehyde showed a fumigant activity against J2s and had the highest nematicidal activity among the VOCs. Egg hatching was remarkably inhibited at 10 mg/mL. The average number of the hatched juveniles per egg mass after three days of acetaldehyde treatment was 3.8, whereas the average number of the hatched juveniles per egg mass of the control treatment was 77.6 (Table 4). However, 1 mg/mL acetaldehyde temporarily inhibited egg hatching. Acetaldehyde was more efficacious in lessening the number of the hatched juveniles than MCCC 1A00493 culture treatment. We also observed 100% mortality of the hatched juveniles in MCCC 1A00493 culture treatment and 10 mg/mL acetaldehyde treatment. Therefore, fumigating juveniles and eggs was an effective method to control *M. incognita*.

### 2.5. Chemotaxis of M. incognita toward Ethylbenzene, Dimethyl disulfide, Acetaldehyde, and 2-Butanone

**Figure 4:**
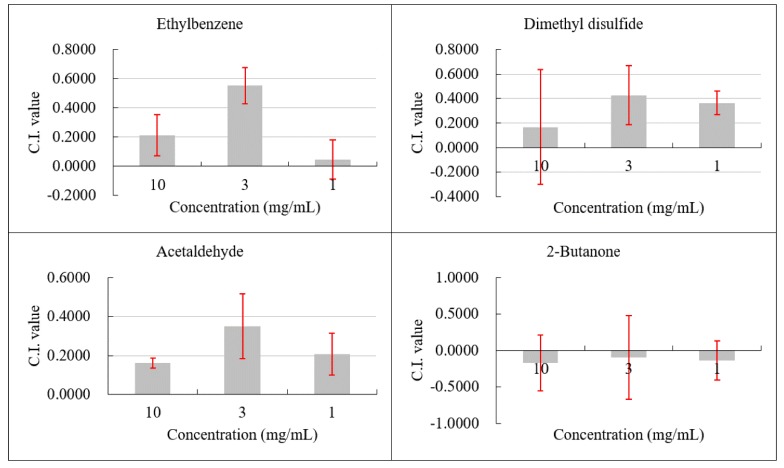
Attracting effect of VOCs on *M. incognita*.

Three volatiles (acetaldehyde, dimethyl disulfide, and ethylbenzene) could attract nematodes moving toward the test solution site. Acetaldehyde treatment had a C.I. of 0.1610, 0.3494, and 0.2065 from high concentration to low concentration; therefore, acetaldehyde showed an attracting activity. Dimethyl disulfide could attract nematodes at 3 and 1 mg/mL with a C.I. of 0.4280 and 0.3651, respectively. Ethylbenzene showed an attracting activity at concentrations of 10 and 3 mg/mL with a C.I. of 0.2137 and 0.5540, respectively. The chemotaxis of 2-butanone was unstable because it had a C.I. fluctuating at 0 and at different concentrations; hence, 2-butanone had a repellent activity (Figure 4). 

## 3. Discussion

Many VOCs that inhibit RKNs are extracted from plant tissues, such as leaves and seeds. Barros et al. [30] studied the nematicidal activity of VOCs emitted by *Brassica juncea*, *Azadirachta indica*, *Canavalia ensiformis*, *Mucunapruriens*, and *Cajanus cajan* against *M. incognita*. Plant VOCs contain diverse molecules that affect the mobility, pathogenicity, and reproduction of *M. incognita*. The nematicidal activity of fresh rucola used for soil amendment in a containerized culture of tomato alleviates nematode infection in a dose-response manner (EC_50_ = 20.03 mg/g) and improves plant growth. The VOCs identified via GC-MS have a strong nematicidal activity against RKNs with low EC_50_ [31]. Studies about nematicidal VOCs from bacteria have been published. VOCs emitted by various bacteria into the chemosphere play a substantial role in antagonistic interactions between microorganisms occupying the same ecological niche and between bacteria and target eukaryotes [32]. Xu et al. identified bacterial VOCs produced by five bacterial strains (*Pseudochrobactrum saccharolyticum*, *Wautersiella falsenii*, *Proteus hauseri*, *Arthrobacter nicotianae*, and *Achromobacter xylosoxidans*). VOCs cover a wide range of aldehydes, ketones, alkyls, alcohols, alkenes, esters, alkynes, acids, ethers, and heterocyclic, and phenolic compounds [33]. 

MCCC 1A00493 produced four kinds of VOCs as identified via GC-MS. In most studies on VOCs of bacteria or fungi, the species of VOCs are generally more than 10 or even over 30. However, only few VOCs were identified in this study. On the one hand, the changes in marine strains under land experimental conditions might not be conducive to the production of their metabolites. On the other hand, some VOCs might not have separated during extraction because of their low content or other factors, such as limitations of technical methods. Consistently, only few VOCs have been identified in marine strains in another study [34]. In the present study, volatiles from the deep-sea bacterial strain *V. dokdonensis* MCCC 1A00493 displayed a strong nematicidal activity against *M. incognita* and had different gas chromatographic profiles. Four main components of the volatiles were identified via GC-MS and their nematicidal activities were confirmed using commercial compounds, thereby making them easily available for the management of RKN disease in the future.

We demonstrated that *V*. *dokdonensis* MCCC 1A00493 showed strong nematicidal activities against RKN through the production of nematicidal volatiles. This study was the first to report the nematicidal volatiles of deep-sea bacteria. Among these VOCs, acetaldehyde and dimethyl disulfide had a contact nematicidal activity. Only acetaldehyde had a fumigation activity against *M. incognita* J2s and could inhibit egg hatching. Acetaldehyde, dimethyl disulfide, and ethylbenzene showed attraction activities, whereas 2-butanone had a weak repellent activity. The C.I. value at 10 mg/mL was lower than that at 3 mg/mL because volatile solutions (acetaldehyde, dimethyl disulfide, and ethylbenzene) with a high concentration of 10 mg/mL might paralyze nematode juveniles and make them move slowly. At 8 h of chemotaxis assay, some of the juveniles moved out from the center of the plate, and some juveniles stayed in the central spot. Another reason was that a high concentration of volatile compounds has a repellent effect on nematode. Few studies on ethylbenzene and 2-butanone have been conducted. Acetaldehyde is widely reported as an insect attractant and insecticide [35] and can be used for the antiseptic and sterilizing effects of fruit and vegetable fumigants [36,37]. Acetaldehyde is a product of ethanol metabolism in vivo and has a certain toxic effect. However, acetaldehyde’s nematicidal activity has yet to be reported. Similar to acetaldehyde, dimethyl disulfide can be used as an insect attractant and insecticide; it is also an effective component in many insecticides and attractants [38,39]. Dimethyl disulfide is a potential biological insecticide for the control of tomato RKN [40]. Dimethyl disulfide had also been reported to have the strongest nematicidal activity (LC90 = 0.162 mmol/L) against *Bursaphelenchus xylophilus* in direct contact for 24 h [34]. Huang et al. [41] found that dimethyl disulfide is active against *M. incognita* juveniles and eggs. Therefore, dimethyl disulfide has been widely studied as a fumigant, which has a good field application effect on RKNs and some fungal diseases. Dimethyl disulfide slightly affects the soil microbial community in the environment. Therefore, this VOC can be used as a soil fumigant instead of bromomethane [42], which has been forbidden for soil fumigation in China since December 30, 2018. 

The toxicity of acetaldehyde and dimethyl disulfide to RKN is unclear. Obvious differences were observed in the morphological characteristics and internal tissues between the untreated RKNs and RKNs treated with acetaldehyde and dimethyl disulfide. The tissues, including the intestine and pharynx, in the cavity of untreated RKNs (Figure 4) are relatively complete. However, in the treated RKNs (Figure 4), only the esophagus and pharynx are complete, and the intestinal tissue in the cavity of the lower part of the worm body is damaged. Further research is needed to understand the molecular mechanisms responsible for contact nematicidal activities. 

Each VOC produced by MCCC 1A00493 has different effects on RKNs. Multiple VOCs can be used to simultaneously control RKNs [43,44]. The mixture of VOCs produced by bacteria may be more effective to control nematodes than treatment with synthetic nematicides composed of a single compound [44]. The VOCs produced by MCCC 1A00493 have the following control modes: direct contact, fumigation, attraction, and repellent activities. Volatile substances are more effective than nonvolatile nematicides in the control of RKNs. RKNs could not be acted upon by nematicides, but VOCs could inhibit or kill RKNs through fumigation or could control RKNs by trapping and repelling, that is, to attract nematodes to move to the application area and kill them. The VOCs of MCCC 1A00493 could kill *M. incognita* J2s through fumigation and inhibit the hatching of its eggs through fumigation. Nematicidal agents could not directly come in contact with eggs on plant roots and juveniles in soils, but these VOCs could effectively inhibit the hatching of nematode eggs. Even few juveniles were inhibited and killed through fumigation. This result showed that VOCs could simultaneously inhibit egg hatching and juvenile development, reduce the population number of RKNs, and control the different development stages of RKNs. Overall, we could establish an integrated strategy or system combining the different functions of nematicidal volatiles on RKNs. The specific mode of action should be further tested in the field.

## 4. Materials and Methods

### 4.1. Bacterial Material

*V. dokdonensis* MCCC 1A00493, which has a strong nematicidal activity, was isolated from polymetallic nodules in the East Pacific Ocean (depth: 4754 m). The bacterium was stored at 4 °C on 2216E medium for a short time. A loop of the fresh culture was transferred into a polyamide bottle containing 10 mL of 2216E medium to produce liquid cultures for the experiments. The liquid cultures were incubated in a rotary shaker (180 r/min) at 28 °C for 20 h as seed liquid. Then, 1 mL of seed fluid was added to a 250 mL conical flask containing 100 mL of 2216E medium and incubated in a rotary shaker (180 r/min) at 28 °C for 48 h. Bacterial culture was used in subsequent experiments.

### 4.2. Chemicals

The following chemicals were used: acetaldehyde (99.5%; Aladdin, shanghai, China); dimethyl disulfide (>98%; TIC Corporation Limited, shanghai, Japan); ethylbenzene (99.5%; Macklin, shanghai, China); 2-butanone (99.0%; Sinopharm Chemical Reagent Company, shanghai, China); and Tween 20 (Biosharp, shanghai, China).

### 4.3. Nematode Population

A population of *M. incognita* was reared on susceptible tomato plants in a greenhouse in State Key Laboratory of Agricultural Microbiology, Wuhan, China, for two months at 25 ± 2 °C. Infested plants were uprooted, and roots with numerous large galls and egg masses were gently washed to remove soil. Egg masses were picked and transferred to 96-well plates, and distilled water was used as a natural hatching agent. The egg masses were incubated at room temperature to obtain juveniles. After hatching, the juveniles were collected, and a suspension of second-stage juveniles (J2s) was prepared in distilled water.

### 4.4. GC-MS Analysis

Solid-phase microextraction (SPME) and gas chromatography–mass spectrometry (GC-MS) were conducted for VOC extraction and analysis [45]. The MCCC 1A00493 was cultured, and its volatiles were collected in accordance with the methods described by Di’az et al. [46]. A new 75 μm carboxen/polydimethylsiloxane fiber (Supelco, Bellefonte, PA, USA) used for SPME was equilibrated with helium at 270 °C for 15 min. Extractions were then performed inside 15 mL Supelco SPME vials filled with 9 mL of bacterial culture or 2216E liquid medium containing a stir bar [34]. An SPME needle was used to pierce the septum, and the fiber was exposed to the headspace of the vial. Extraction was performed at 60 °C for 1 h with constant magnetic stirring. The volatiles from the 2216E liquid medium were used as control. Each sample was tested three times.

After extraction, the fiber was directly inserted into the injection port of the GC-MS instrument (Hewlett-Packard (HP) 7890A-5975C, Agilent Technologies, USA) and desorbed at 270 °C for 2 min. The GC/MS instrument was equipped with a DB-5MS capillary column (30 m × 0.25 mm × 0.25 μm). The carrier gas was helium with a flow rate of 1 mL/min. The program used to control the temperature of the oven was 40 °C for 2 min, 40 °C–180 °C at a rate of 4 °C/min, 180 °C–240 °C at 5 °C/min, and held at 240 °C for 6 min. The temperatures of the transfer line and ion trap were 150 °C and 250 °C, respectively. The volatile compounds were identified from the database search through the comparison of their mass spectrum with the GC/MS system data bank of the National Institute of Standards and Technology (NIST 08).

### 4.5. Nematicidal Activity Bioassays of the Fermentation Supernatant and VOCs of MCCC 1A00493

The fermentation supernatant of MCCC 1A00493 was prepared. VOCs were also prepared in distilled water containing Tween 20, and the final concentration of Tween 20 in the treatment was 0.3%.

The contact nematicidal activity of the fermentation supernatant or VOCs of MCCC 1A00493 was tested. The sample (100 μL) was added to one well in a 96-well plate with a nematode suspension containing about 60 worms. The plates were immediately wrapped with Parafilm. After incubation at room temperature (20–25 °C), the mobile (live) and immobile juveniles were observed under an inverted microscope (Olympus, IX73). *M. incognita* was considered dead when no movement was observed for 2 s after it was touched with a needle. Mortality values were corrected by eliminating natural death in a negative control in accordance with Schneider–Orelli’s formula [47], which is expressed as follows:

Mortality = [(mortality percentage in treatment − mortality percentage in control) /(1 − mortality percentage in control)] × 100%.(1)

### 4.6. Fumigant Activity of the Fermentation Supernatant and VOCs of MCCC 1A00493 against M. incognita Juveniles

The fumigant activity of the fermentation supernatant and VOCs of MCCC 1A00493 was tested [48] by adding 200 μL of the fermentation supernatant or VOC solution to one well in the center of a 96-well plate. The concentration of VOCs was set as 10 mg/mL, and nematode juveniles with 100 μL of ddH_2_O were added to the four wells adjacent to the test sample. The nematodes were prepared as above. The plates were immediately wrapped with Parafilm to prevent the escape of the volatiles. After incubation at room temperature (20–25 °C) for 24 h, the mobile (live) and immobile juveniles were observed under an inverted microscope (Olympus, IX73).

### 4.7. Fumigant Activity of VOCs to Inhibit Egg Hatching

The method used in this experiment was similar to the test of fumigant activity to juveniles. Egg masses were collected from the infested roots and washed with ddH_2_O to remove soil. The nematodes in each well were replaced with a single egg mass, which was hatched in distilled water and used as control. The number of hatched worms was counted under the microscope after 1, 2, and 3 days. Each treatment was repeated three times. 

### 4.8. Chemotaxis Test

**Figure 5:**
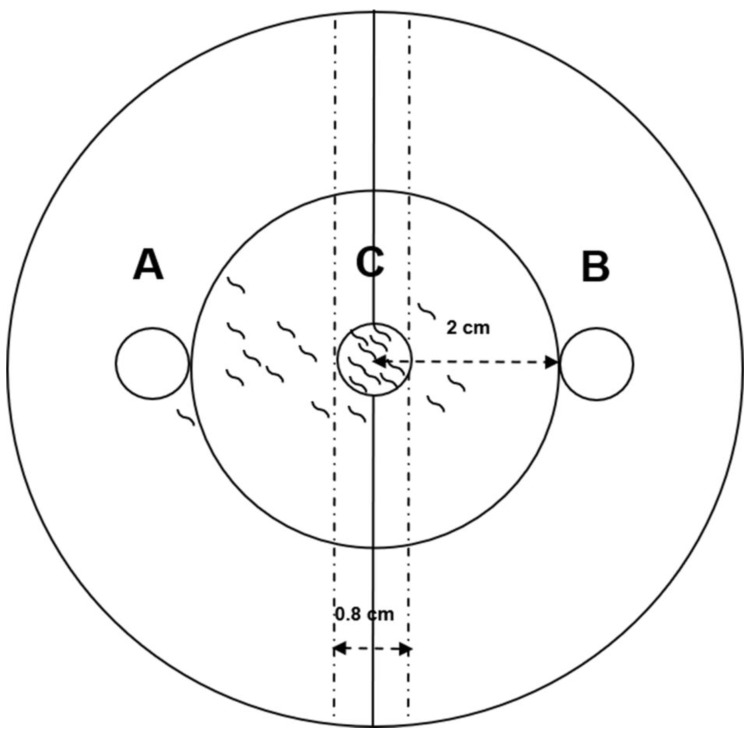
Chemotaxis mode. (**A**) Test location, (**B**) control location, and (**C**) center of the plate.

A chemotaxis mode (Figure. 5) was designed in accordance with previously described methods with some modifications [49,50]. About 100 juveniles were placed in the center of 2% water agar plate. A volatile compound solution (30 μL of 10, 3, and 1 mg/mL) was added to a round paper (1 cm^2^) 4 cm away from the center of the assay plate. Tween-20 (30 μL) was added to the paper on the opposite side of the VOC solution as control.

The number of worms at the test location or the control location was counted under an inverted microscope after incubation in the assay plate at 20 °C for 8 h. The juveniles that remained within 0.4 cm of the midline were not counted for chemotaxis assays. The chemotaxis index (C.I.) was finally calculated with the following formula [51]:

C.I. = (the number of worms at the test location − the number of worms at the control location)/total number of worms on the plate.

A C.I. between 0 and 1 means an attracting activity, whereas a C.I. between −1 and 0 means a repellent activity.

The experiment was repeated twice and carried out in triplicate (Figure 5).

### 4.9. Data Analysis and Atatistics

Data were analyzed by using ANOVA, and means were compared through least significant differences (Duncan) at *p* = 0.05 by using SPSS 17.0 for Windows.

## 5. Conclusions

In summary, we isolated four kinds of volatiles (acetaldehyde, 2-butanone, dimethyl disulfide, and ethylbenzene) produced from *V. dokdonensis* MCCC 1A00493. The four VOCs had a variety of active modes on *M. incognita* juveniles. Acetaldehyde had direct contact killing, fumigation, and attraction activities; dimethyl disulfide had direct contact killing and attraction activities, ethylbenzene had an attraction activity; and 2-butanone had a repellent activity. Only acetaldehyde had a fumigant activity to inhibit egg hatching. Combination of direct contacted killing and attraction make nematicidal agents can trap and kill nematodes. Nematicidal volatiles also could kill nematode and inhibit egg hacthing by fumigation. *V. dokdonensis* MCCC 1A00493 control nematodes through multi-mode, multi-function integrated strategy. This suggests that bacterial volatiles represent an important source for new natural nematicidal compounds that may be developed as novel nematicidal agents. And an integrated strategy which was more effective than current method could be established to control RKNs. 

## Figures and Tables

**Table 1:** Area percentage of the VOCs of the culture of *V. dokdonensis* MCCC 1A00493 through CAR/DVB extraction.

PK	RT	Area Pct	Library/ID	CAS
1	1.6566	17.7158	Acetaldehyde	000075-07-0
2	2.4153	3.1635	2-Butanone	000078-93-3
3	4.4139	2.288	Dimethyl disulfide	000624-92-0
4	7.9255	0.977	Ethylbenzene	000100-41-4
5	9.7593	21.4172	2,5-Dimethyl pyrazine	000123-32-0
6	11.5195	19.0497	Benzaldehyde	000100-52-7

**Table 2:** Contact nematicidal activity of the VOCs against *M. incognita*.

Compound	EC_50_ (mg/L)
	24 h
Acetaldehyde	<3
Dimethyl disulfide	139.1
2-Butanone	>1000
Ethylbenzene	>1000

**Table 3:** Fumigant activity of VOCs against *M. incognita*.

	Mortality (%) ± SD	
	6 h	24 h
10 mg/mL acetaldehyde	100	100
1 mg/mL acetaldehyde	70.0 ± 12.0	97.9 ±2.4
10 mg/mL 2-butanone	0	1.4 ± 1.9
10 mg/mL dimethyl disulfide	0	7.9 ± 1.6
10 mg/mL ethylbenzene	0	1.3 ± 1.6

**Table 4:** Effect of acetaldehyde and bacterial culture to *M. incognita*’s egg hatching.

	Hatched Worms Per Egg Mass ± SD		
	1 day	2 days	3 days
1 mg/mL acetaldehyde	17.3 ± 7.0 b	37.4 ± 13.3 ab	77.1 ± 6.0 a
10 mg/mL acetaldehyde	3.7 ± 2.3 b	3.8 ± 2.3 c	3.8 ± 2.3 b
1A00493 culture	18.7 ± 8.8 b	18.9 ± 8.7 bc	19.6 ± 8.4 b
control	43.9 ± 21.0 a	62.4 ± 23.7 a	77.6 ± 25.7 a

The number represents the means of the replicates ± SD. Experimental data were analyzed using SPSS 17.0. Comparison between groups was analyzed through single-factor ANOVA. Different lowercase letters indicate significant difference between treatments (*p* < 0.05).
